# The Three-Class Annotation Method Improves the AI Detection of Early-Stage Osteosarcoma on Plain Radiographs: A Novel Approach for Rare Cancer Diagnosis

**DOI:** 10.3390/cancers17010029

**Published:** 2024-12-25

**Authors:** Joe Hasei, Ryuichi Nakahara, Yujiro Otsuka, Yusuke Nakamura, Kunihiro Ikuta, Shuhei Osaki, Tamiya Hironari, Shinji Miwa, Shusa Ohshika, Shunji Nishimura, Naoaki Kahara, Aki Yoshida, Tomohiro Fujiwara, Eiji Nakata, Toshiyuki Kunisada, Toshifumi Ozaki

**Affiliations:** 1 Department of Medical Information and Assistive Technology Development, Graduate School of Medicine, Dentistry and Pharmaceutical Sciences, Okayama University, Okayama 700-8558, Japan; 2 Science of Functional Recovery and Reconstruction, Graduate School of Medicine, Dentistry and Pharmaceutical Sciences, Okayama University, Okayama 700-8558, Japan; 3 Department of Radiology, Juntendo University School of Medicine, Tokyo 113-8431, Japan; 4 Milliman, Inc., Tokyo 102-0083, Japan; 5 Plusman LCC, Tokyo 103-0023, Japan; 6 Department of Orthopedic Surgery, Graduate School of Medicine, Nagoya University, Nagoya 464-0083, Japan; 7 Department of Musculoskeletal Oncology and Rehabilitation, National Cancer Center Hospital, Tokyo 104-0045, Japan; 8 Department of Musculoskeletal Oncology Service, Osaka International Cancer Institute, Osaka 541-8567, Japan; 9 Department of Orthopedic Surgery, Kanazawa University Graduate School of Medical Sciences, Ishikawa 920-8641, Japan; 10 Department of Orthopaedic Surgery, Hirosaki University Graduate School of Medicine, Aomori 036-8563, Japan; 11 Department of Orthopaedic Surgery, Kindai University Hospital, Osaka 589-8511, Japan; 12 Department of Orthopedic Surgery, Mizushima Central Hospital, Kurashiki 712-8064, Japan

**Keywords:** osteosarcoma, medical image annotation, anatomical annotation method, rare cancer

## Abstract

**Background/Objectives**: Developing high-performance artificial intelligence (AI) models for rare diseases is challenging owing to limited data availability. This study aimed to evaluate whether a novel three-class annotation method for preparing training data could enhance AI model performance in detecting osteosarcoma on plain radiographs compared to conventional single-class annotation. **Methods**: We developed two annotation methods for the same dataset of 468 osteosarcoma X-rays and 378 normal radiographs: a conventional single-class annotation (1C model) and a novel three-class annotation method (3C model) that separately labeled intramedullary, cortical, and extramedullary tumor components. Both models used identical U-Net-based architectures, differing only in their annotation approaches. Performance was evaluated using an independent validation dataset. **Results**: Although both models achieved high diagnostic accuracy (AUC: 0.99 vs. 0.98), the 3C model demonstrated superior operational characteristics. At a standardized cutoff value of 0.2, the 3C model maintained balanced performance (sensitivity: 93.28%, specificity: 92.21%), whereas the 1C model showed compromised specificity (83.58%) despite high sensitivity (98.88%). Notably, at the 25th percentile threshold, both models showed identical false-negative rates despite significantly different cutoff values (3C: 0.661 vs. 1C: 0.985), indicating the ability of the 3C model to maintain diagnostic accuracy at substantially lower thresholds. **Conclusions**: This study demonstrated that anatomically informed three-class annotation can enhance AI model performance for rare disease detection without requiring additional training data. The improved stability at lower thresholds suggests that thoughtful annotation strategies can optimize the AI model training, particularly in contexts where training data are limited.

## Simple Summary

Developing effective artificial intelligence (AI) systems for rare diseases such as osteosarcoma is challenging owing to the limited available data. This study introduces a novel approach for preparing training data for AI systems that detect osteosarcoma using X-rays. Traditional methods label tumor areas as a single entity; however, our new approach divides tumor regions into three distinct classes: intramedullary, cortical, and extramedullary. This three-class annotation method enables AI systems to learn more effectively from limited datasets by incorporating detailed anatomical knowledge. This new approach to data preparation resulted in more robust AI models that could detect subtle tumor changes at lower threshold values, demonstrating how strategic data annotation methods can enhance AI performance even with limited training samples. This methodological innovation in data preparation offers a new paradigm for developing AI systems for rare diseases, for which traditional data-driven approaches often fall short.

## 1. Introduction

Primary malignant bone tumors, despite their extremely low incidence [1,2,3], predominantly affect children, adolescents, and young adults and require comprehensive support measures, including psychosocial care [4,5,6]. Diagnostic delays, owing to the rarity of these tumors, can significantly impact patient prognosis. Early detection is crucial for improving patient outcomes; however, their rarity often leads to diagnostic delays, particularly among adolescents and young adults, where these conditions are frequently misdiagnosed as growing pain or exercise-related disorders [7]. The nonspecific nature of the initial symptoms, combined with the challenges that general orthopedic surgeons face in making accurate initial diagnoses, compounds this problem.

Primary screening using radiographic imaging plays an important role in the diagnosis of primary malignant bone tumors. However, detecting subtle changes in bone structure requires considerable expertise, and even experienced specialists face the risk of oversight. Furthermore, disparities in access to specialist care across regions present additional barriers to early diagnosis [8,9,10].

Therefore, the development of artificial intelligence (AI)-assisted diagnostic imaging systems has attracted considerable attention. Recent advances in AI for medical imaging have demonstrated transformative potential across multiple applications, ranging from image segmentation and structure identification to automated abnormality detection and predictive analytics. In particular, deep learning-based methods have shown improved performance in oncologic imaging tasks, including tumor characterization and treatment response prediction, thereby contributing to better patient outcomes [11]. Currently, two main AI-based approaches are widely used in medical imaging: traditional machine learning (ML) algorithms that learn from predefined features such as texture and morphology and deep learning methods utilizing complex neural networks that can automatically extract and optimize meaningful parameters directly from raw image data [11]. These approaches offer enhanced sensitivity, specificity, and reliability in radiological evaluations and are of special interest in the context of rare diseases, where data scarcity has historically limited the effectiveness of conventional diagnostic methods. Advances in ML technology have enabled the construction of AI models capable of learning features from large volumes of medical imaging data and detecting abnormalities with high precision [12,13]. Nevertheless, for rare conditions such as primary malignant bone tumors, the limited availability of training data poses significant challenges. Moreover, conventional data-driven approaches have limitations in development under such data-scarce conditions [14].

Previous studies on AI development for X-ray image interpretation of primary malignant bone tumors have solely utilized models trained on unannotated data, achieving notably limited performance, with sensitivity rates of approximately 70%, showing minimal improvement in recent years [15,16,17]. Thus, in the interpretation of X-ray images of primary malignant bone tumors, the handling of training data may be more crucial than the selection of baseline models.

Unlike in MRI, abnormal shadows on X-ray images of primary malignant bone tumors are often extremely subtle and difficult to detect, making this a particularly challenging task. Although various reports exist on MRI image interpretation models [18,19,20], studies on X-ray image interpretation remain scarce. Additionally, while MRI and CT scans can yield multiple images per case, X-ray imaging provides fewer images, making it particularly challenging to accumulate substantial image data for rare conditions, such as primary malignant bone tumors. Consequently, creating high-quality datasets is crucial for improving model performance.

Therefore, we focused on developing high-quality annotated data as a novel approach to maximize the utility of limited data. Previously, through detailed tumor region segmentation performed by bone and soft tissue tumor specialists while referencing MRI images, we successfully developed high-performance AI models by creating annotated data that capture subtle changes typically difficult to discern in X-ray images alone [21].

In this study, we report the development of a novel methodology called the “three-class annotation method” for annotating primary malignant bone tumor image data, which enabled us to further enhance the performance of X-ray image interpretation AI models using the same dataset. Herein, we present the procedures and effectiveness of the proposed approach. This study addressed the critical challenges in rare disease diagnosis through AI, focusing on enhancing model performance with limited datasets through anatomically informed annotation strategies. Our findings demonstrated that strategic annotation methods can significantly improve diagnostic capabilities, even with limited data availability, offering a new paradigm for AI development in rare cancer diagnosis.

## 2. Materials and Methods

### 2.1. Study Population and Dataset

This study utilized 468 X-ray images from 31 patients with osteosarcoma of the distal femur who were treated at Okayama University Hospital. All patients were pathologically confirmed to have osteosarcoma. Of these images, 328 were used for training. For the normal knee images, 265 of the 378 images from 54 patients without degenerative changes were used for training. The remaining images were used for validation. Given the rarity of osteosarcoma, developing models with limited image datasets necessitated the adoption of strategies to maximize the image diversity in each case. Considering the constraints of the available cases, we implemented a unique approach to partition the dataset into training and internal validation subsets. To optimize the model generalization capability and diagnostic accuracy by maximizing the image diversity in both subsets, we pooled all available cases and randomly selected 70% of the images for training, with the remaining 30% designated for internal validation. Although this methodology resulted in images from the same patients appearing in both the training and validation sets, potentially leading to overfitting, it was chosen as a strategic tradeoff to mitigate the limitations imposed by the small number of available cases. To address potential overfitting concerns, we employed a completely independent dataset for the final performance evaluation, enabling the assessment of the model’s generalization capabilities using unpublished data from different institutions.

### 2.2. Image Annotation Methods

**Figure 1:**
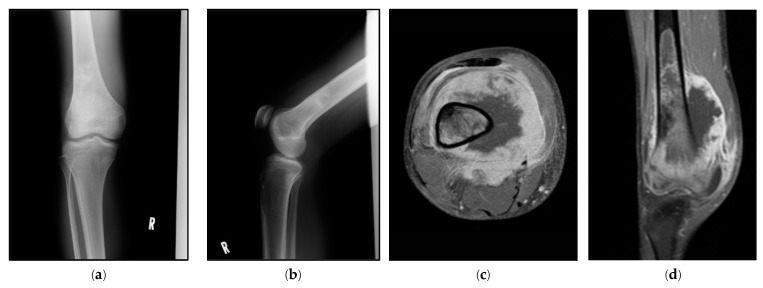

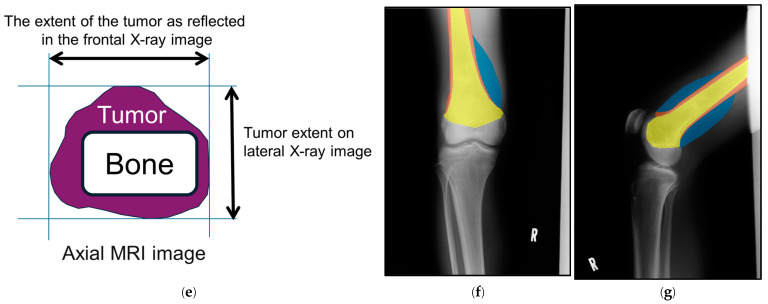
Radiographic images showing the process of tumor segmentation and annotation using both X-ray and MRI guidance. This figure demonstrates our systematic approach to tumor segmentation, in which MRI images provide crucial guidance for the accurate annotation of tumor boundaries on X-ray images, which alone may not clearly reveal the full extent of the lesion: (**a**,**b**) anteroposterior (AP) and lateral X-ray views of the distal femur showing subtle bone changes, demonstrating the challenge of determining tumor boundaries on plain radiographs; (**c**,**d**) axial and sagittal T1-weighted fat-suppressed contrast-enhanced MRI images clearly delineating the tumor extent; (**e**) schematic diagram illustrating how the tumor extent visible on axial MRI correlates with the measurements on AP and lateral X-ray projections; (**f**,**g**) annotated AP and lateral X-ray images utilizing the MRI-guided three-class annotation method, where yellow indicates the intramedullary tumor component, orange represents cortical bone involvement, and blue indicates extramedullary tumor extension.

Two distinct annotation methodologies were applied to the same dataset. The first approach annotated the tumor region as a single class (1C method), whereas the second approach divided the tumor into three classes (3C method). Primary malignant bone tumors are malignant neoplasms that originate within the bone and exhibit extraosseous extension. These tumors can spread through the Haversian canals and may manifest notable periosteal reactions such as Codman’s triangle [22]. This results in distinct clinical radiographic findings in the intraosseous and extraosseous regions. Therefore, we aimed to enhance the model performance by separately annotating tumors in three distinct regions: intraosseous, cortical, and extraosseous zones. The 3C annotation method does not divide the dataset into separate categories but rather annotates each tumor image into three distinct anatomical regions: intramedullary, cortical, and extramedullary. This approach enriches the training data by providing detailed anatomical information within each image, allowing the model to learn region-specific features more effectively. The segmentation was performed manually by a single author with over 15 years of experience in the diagnosis and treatment of musculoskeletal tumors. This expert leveraged both MRI and X-ray images to ensure accuracy and consistency in identifying anatomical boundaries. MRI images provided superior contrast for delineating soft tissue and intramedullary structures, while X-ray images offered complementary anatomical information. The segmentation regions were then validated by another board-certified orthopedic surgeon. The intramedullary tumor component is annotated in yellow, cortical bone involvement in orange, and extramedullary tumor extension in blue (Figure 1).

### 2.3. Network Architecture and Model Training Process

In this study, we employed the standard U-Net architecture as originally described by Ronneberger et al. (2015) [23] without incorporating additional modifications or enhancements. Specifically, we did not implement variations such as attention mechanisms, residual connections, or other advanced modules. The primary reason for this choice was to evaluate the effectiveness of the proposed three-class annotation method in isolation, without the confounding effects of architectural complexities. Furthermore, transfer learning was not utilized. Instead, the model was trained from scratch using the annotated dataset prepared for this study. This decision was made to ensure that the results reflect the direct impact of our annotation methodology rather than pre-trained features from unrelated tasks or datasets. Pixel deshuffling was applied before the first convolution to reduce the total number of network parameters, and pixel shuffling was performed after the final convolution. Convolutional operations were conducted for each unit, referred to in this study as a “Res block”. The Res block features an architecture similar to that of a residual block [24] with a skip connection. The Res block is designed to enhance feature propagation and mitigate the vanishing gradient problem, thereby improving the stability of deeper networks. The network output utilizes multiresolution maps in the same manner as the U-Net. Each X-ray image was first renormalized using a window level within the 1^st^–99^th^ percentile of the pixel values to exclude outliers. This step ensures consistent pixel intensity across images, which is critical for effective feature learning. The images were then resampled into 1 mm squares in the imager pixel spacing space. During training, 128 mm square patches were cropped to include a lesion area with a 0.5 ratio and augmented through random intensity shifts (probability = 0.5, offsets = 0.05) and random affine transformations (probability = 0.5) using Project MONAI (version 1.3.1) [25]. These augmentation techniques were chosen to enhance model generalization and robustness against limited data availability. The model was trained in an end-to-end manner using a stochastic gradient descent optimizer with a cross-entropy loss. We employed the One Cycle LR [26] with a maximum learning rate of 1 × 10^−1^ and completed a total of 100 steps over 40 cycles. At the end of each epoch, the DICE coefficient [27] for the entire annotated tumor region was calculated to identify the best metric epoch. The DICE coefficient serves as an evaluation metric that assesses the accuracy of the AI-segmented region compared to the ground-truth-annotated tumor region. To evaluate the performance of the diagnostic tool in detecting lesions, we calculated the sensitivity, specificity, and DICE coefficient and determined the optimal cutoff value for lesion detection probability. The entire program was developed using Python 3.11 with Pytorch 2.0.1 (Python Software Foundation). All analyses were conducted using Pytorch, sklearn, and scipy packages. All patient data utilized in this study were obtained retrospectively, and patient consent was obtained via the opt-out method. The participants were allowed to withdraw from the study at any point. This study was approved by the institutional ethics committee and conducted following all ethical guidelines to ensure patient privacy and transparent data usage.

### 2.4. Model Evaluation Process

For the performance evaluation, we analyzed 268 X-ray images from 32 cases of distal femoral osteosarcoma from the Osaka International Cancer Center and 554 normal knee X-ray images from 148 cases from Mizushima Central Hospital as evaluation data. The evaluation dataset contained no images used during training. In this study, we employed receiver operating characteristic (ROC) curve analysis to determine the optimal cutoff value for evaluating the performance of the imaging equipment. Initially, we calculated sensitivity and specificity scores using an image dataset labeled as abnormal (label “1”) and normal (label “0”). These sensitivity and specificity values were then used to generate the ROC curves. The ROC curve plots sensitivity (true-positive rate) on the vertical axis against one-specificity (false-positive rate) on the horizontal axis, and the cutoff value was determined using the Youden index (J) [28]. We calculated the Youden index for cutoff values ranging from 0 to 1 in increments of 0.001 for each equipment’s scores and determined the optimal cutoff as the value that maximized the Youden index. This approach represents an effective methodology for maximizing the classifier performance while maintaining a balance between sensitivity and specificity [29]. Additionally, we calculated the area under the curve (AUC) to evaluate the overall classifier performance. For the calculation of the “score”, which represents the AI’s determination, we employed the softmax function to compute probabilities for each class. The softmax function implemented in the final output layer of the neural network converts the class scores into probability distributions. Specifically, we calculated the probability of belonging to each class using the following equation, where *zi* represents the output from the final layer:

$$\mathrm{Score}=\mathrm{softmax}(\mathrm{zi})=\frac{exp(zi)}{\sum_{j=1}^{K}exp(zj)}$$

Here, *zi* represents the *i*-th output from the final layer, and *K* denotes the total number of classes. The scores calculated using this softmax function were interpreted as the probabilities of belonging to each class and were utilized as the model’s predictive output. This methodology enabled the comparison of scores between different classes and allowed the model to perform classifications with confidence levels for each class. Furthermore, this approach facilitated the appropriate evaluation of class scores in the calculation of ROC curves and AUC values.

### 2.5. Statistical Analysis

For a comparative evaluation of the diagnostic performances between the 1C and 3C models, we conducted statistical analyses focusing on sensitivity and specificity. Sensitivity (true-positive rate) was defined as the proportion of actual positives correctly identified by the model and specificity (true-negative rate) as the proportion of actual negatives correctly identified. To determine whether there were statistically significant differences in the sensitivity and specificity between the two models, we employed McNemar’s test. This test is appropriate for paired nominal data, allowing the assessment of differences in classification outcomes for the same subjects between the two models. McNemar’s test was selected based on its suitability for evaluating paired dichotomous outcomes, particularly for diagnostic test comparisons [30]. Additionally, we calculated the 95% confidence intervals (Cis) for sensitivity and specificity using the Wilson score method [31], providing a measure of the precision of our estimates. Statistical significance was set at *p* < 0.05. This study utilized the same patient cohort as in our previous study [21], which demonstrated the effectiveness of expert annotations in AI-based osteosarcoma detection. Building on these findings, we developed a novel three-class annotation method to further enhance the model performance. For detailed patient demographics and clinical characteristics, the readers can refer to our previous publication. This test is appropriate for paired nominal data, specifically in our case where two different annotation methods (1C and 3C) were applied to the same dataset, allowing for the assessment of differences in classification outcomes for the same subjects between the two models.

## 3. Results

### 3.1. Comparison of Overall Model Performance

**Figure 2:**
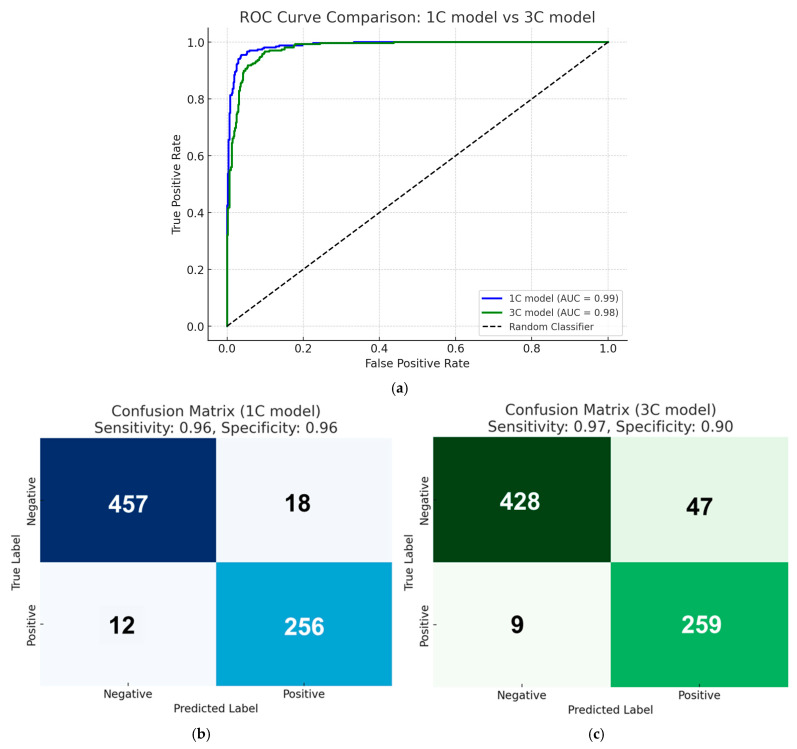
Comparison of the performance of the 1C model and the 3C model for image classification tasks: (**a**) ROC curve comparison between the 1C model (blue line) and the 3C model (green line). The area under the curve (AUC) for the 1C model is 0.99, while the AUC for the 3C model is 0.98. The dashed diagonal line represents the performance of a random classifier (AUC = 0.50); (**b**) the confusion matrix for the 1C model. The 1C model achieved a sensitivity of 96% and a specificity of 96%. The matrix shows the number of true negatives (457), false positives (18), false negatives (12), and true positives (256); (**c**) the confusion matrix for the 3C model. The 3C model achieved a sensitivity of 97% and a specificity of 90%. The matrix shows the number of true negatives (428), false positives (47), false negatives (9), and true positives (259).

In this study, we compared the performance of 1C and 3C models. The analysis revealed several distinct characteristics between the two models. Regarding the diagnostic accuracy (scores), the 1C model yielded a mean score of 0.954 (standard deviation 0.137) versus 0.771 (standard deviation 0.270) of the 3C model. In the discrimination performance evaluation using ROC curves and AUC, the 1C model achieved an AUC of 0.99, and the 3C model achieved 0.98, confirming the superior discriminative ability of both models (Figure 2a). The optimal cutoff values balancing sensitivity and specificity were as follows: the 1C model’s optimal cutoff value was 0.769, whereas the 3C model showed a significantly lower value of 0.131. At these points, the 1C model demonstrated a sensitivity of 95.52% and specificity of 96.21%, while the 3C model showed a sensitivity of 96.64% and specificity of 90.11%. While there was no statistically significant difference in sensitivity, the specificity showed a statistically significant difference (*p* < 0.05). However, the 3C model maintained a satisfactory specificity of >90% (Figure 2b,c).

### 3.2. Analysis of Model Sensitivity and Specificity Patterns

**Figure 3:**
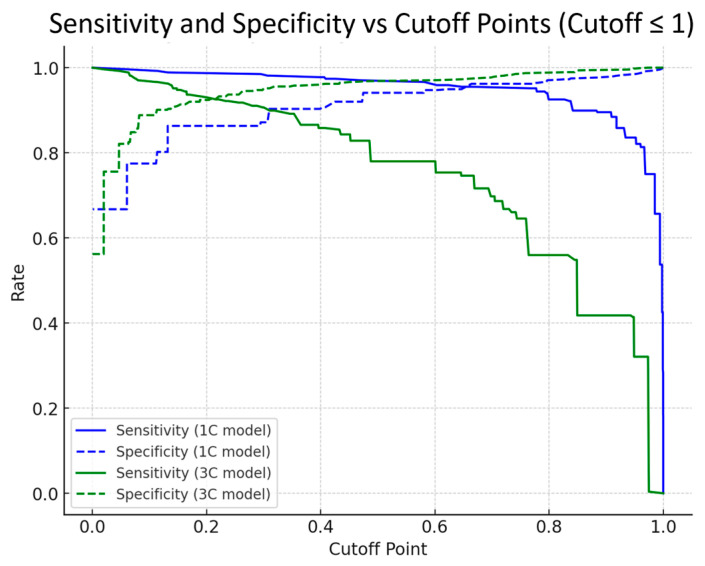
Sensitivity and specificity curves plotted against cutoff values for the 1C and 3C models. The graph displays sensitivity (solid lines) and specificity (dashed lines) values on the *y*-axis (rate) against cutoff values (0.0 to 1.0) on the *x*-axis. The blue lines represent the 1C model’s performance metrics (sensitivity: solid blue line; specificity: dashed blue line), while the green lines show the 3C model’s performance metrics (sensitivity: solid green line; specificity: dashed green line). The values range from 0.0 to 1.0 on both axes, with grid lines marking intervals of 0.2.

Further analysis of sensitivity and specificity changes across continuous cutoff variations revealed characteristic differences between the models (Figure 3). The 3C model consistently maintained higher specificity than the 1C model. While the 1C model required higher cutoff values to achieve high specificity, it showed a sharp decline in sensitivity beyond its optimal cutoff of 0.769. In contrast, the 3C model demonstrated a stable sensitivity–specificity balance at lower cutoff values, confirming its ability to reliably detect abnormalities even at a low cutoff. When setting the score cutoff value at 0.2, the 1C model showed a specificity of 83.58% and sensitivity of 98.88%, while the 3C model demonstrated more balanced results, with a specificity of 92.21% and sensitivity of 93.28%. At the 25th percentile cutoff values (1C model: 0.985, 3C model: 0.661), both models showed identical false-negative counts of 67, indicating that the 3C model could detect abnormalities at lower scores while maintaining a sensitivity comparable to that of the 1C model.

### 3.3. Evaluation of Abnormal Region Detection Capability

In evaluating the abnormal region capture capability using DICE coefficients, the 1C model averaged 0.663 (standard deviation 0.243) and the 3C model averaged 0.644 (standard deviation 0.247), showing no statistically significant difference (*p* = 0.380). This confirms that the 3C model can capture abnormal regions with an accuracy equivalent to that of the 1C model.

### 3.4. Correlation Analysis

**Figure 4:**
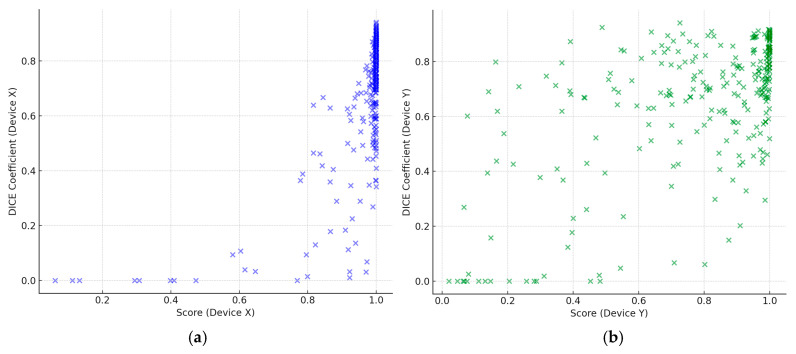
Scatter plots depicting the relationship between the score and DICE coefficient for the 1C and 3C models: (**a**) the relationship between the score (on the *x*-axis) and the DICE coefficient (on the *y*-axis) for the 1C model. The correlation coefficient between the score and the DICE coefficient is 0.682; (**b**) the relationship between the score (on the *x*-axis) and the DICE coefficient (on the *y*-axis) for the 3C model. The correlation coefficient between the score and the DICE coefficient is 0.594.

The correlation analysis between the scores and DICE coefficients showed moderate correlations for both models, with correlation coefficients of 0.682 for the 1C model and 0.594 for the 3C model (Figure 4). These results indicate that while both models maintain equivalent sensitivity and abnormal region capture capabilities with very high specificity, the 3C model demonstrates the ability to detect abnormalities at lower scores than those of the 1C model.

## 4. Discussion

### 4.1. Comparative Analysis of Model Performance

**Figure 5:**
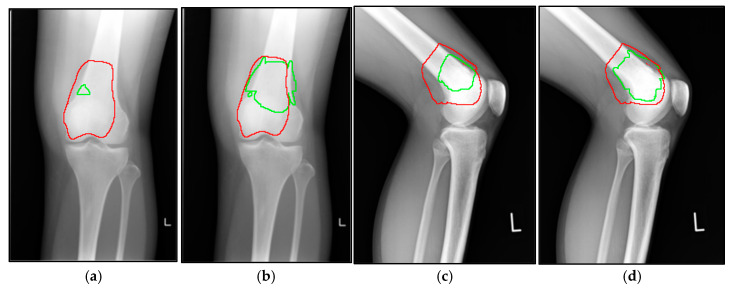
Comparison of lesion detection performance between 1C and 3C AI models in identifying osteosarcoma on X-ray images: (**a**,**b**) anteroposterior radiographs showing osteosarcoma detection results of (**a**) 1C model and (**b**) 3C model outcomes, where the red line represents the osteosarcoma area (as determined by expert human annotation), and the green line indicates the lesion area identified by the AI model; (**c**,**d**) lateral radiographs showing the same comparison, namely (**c**) 1C model and (**d**) 3C model outcomes, with red lines representing expert human annotation and green lines showing AI model detection. “L” indicates the left side of the patient.

The three-class annotation method AI model (3C model) for X-ray image interpretation developed in this study demonstrated distinctive characteristics compared with the conventional single-class annotation method (1C model), introducing a novel perspective in image data preparation for primary malignant bone tumor X-ray interpretation models. This is the first study to demonstrate how AI model characteristics can be altered by annotating a single tumor into multiple classes based on clinically distinct findings. In the conventional single-class annotation method, treating the entire tumor as a single class results in mixed information from regions showing different X-ray characteristics, such as extraosseous tumors, intraosseous tumors, and cortical regions. When the cutoff was set based on the 25th percentile value, this approach showed very high specificity (99.16%) but required an extremely high cutoff value (0.985), suggesting the potential oversight of subtle abnormalities or early-stage tumors with lower scores. Conversely, the three-class annotation method introduced in this study enabled the AI model to learn the detailed characteristics of each region by separating the tumor annotations into three distinct areas. Consequently, when the cutoff was set based on the 25th percentile value, the model could detect abnormalities at a lower cutoff value (0.661), proving it to be more suitable for screening purposes, which is the most critical application of this model. Comparing the performance of both models, while the 1C model (mean 0.954) outperformed the 3C model (mean 0.771) in terms of diagnostic accuracy scores, there was no statistically significant difference in abnormal region capture capability, evaluated using DICE coefficients (1C model: 0.663, 3C model: 0.644, *p* = 0.380). This suggests that the three-class annotation method can provide more detailed information without compromising the detection accuracy. Indeed, in cases where intraosseous tumor shadows were particularly subtle, the 3C model demonstrated a more accurate tumor range capture (Figure 5).

### 4.2. Theoretical Framework and Mechanisms

The impact of this three-class annotation method on the AI model learning processes can be considered from multiple theoretical perspectives. First, from the viewpoint of feature space partitioning and representation learning, although the 1C model recognized the entire tumor region as a single feature space with mixed image characteristics, the 3C model enabled clearer learning of image features specific to each anatomically distinct region (intraosseous, cortical, and extraosseous). This allowed for a more detailed recognition of the differences between normal structures and pathological changes in each region. Second, the 3C model could effectively perform multi-task learning, enhancing the model generalization capability through parallel learning of recognition in each region. Learning inter-regional relationships enables the acquisition of more robust feature representations, which is particularly advantageous when learning from limited datasets, such as in this study. Furthermore, the 3C model formed independent decision boundaries for each region, enabling finer discrimination. The presence of multiple decision boundaries enables more flexible abnormality detection, providing theoretical grounds for the high detection capability at low cutoff values observed in this study.

### 4.3. Clinical Implications and Applications

From a clinical application perspective, the characteristic differences between the models have significant implications. The 1C model represents a “certainty-focused” approach that detects only clear abnormalities with high confidence, while the 3C model represents a “flexibility-focused” approach suitable for initial screening and follow-up monitoring; thus, it is capable of evaluating a broader range of abnormalities incrementally. Particularly noteworthy is the potential contribution of the 3C model’s lower cutoff value to the early detection of primary malignant bone tumors, which is a rare cancer type. This implies an increased possibility of detecting subtle changes in the early stages, potentially leading to more opportunities for early diagnosis and appropriate therapeutic intervention. Setting the 1C model’s cutoff at 0.985 poses clinical challenges owing to its extremely high value, potentially flagging normal physiological variations or minor image noise as abnormal. This could lead to unnecessary examinations and treatments and increased patient burden, potentially overconsuming medical resources. For instance, when setting the cutoff of both models at 0.2, the 1C model showed 98.88% sensitivity and 83.58% specificity, whereas the 3C model demonstrated 93.28% sensitivity and 92.21% specificity, confirming the 3C model’s capability for balanced diagnosis. While the 1C model could be used with a low cutoff value when prioritizing sensitivity, considering the need to reduce false positives, the 3C model appeared to be more appropriate. At a 0.2 cutoff, the false-positive rates were 16.42% for the 1C model and 7.79% for the 3C model, indicating the 3C model’s superior suitability for clinical use. These theoretical characteristics provide a foundation for explaining the experimental results demonstrated by the 3C model, particularly its high detection capability at lower cutoff and more stable specificity maintenance. This theoretical framework also suggests potential applications in other medical image diagnostic tasks.

### 4.4. Future Directions and Challenges

The limitations of this study include the scale and diversity of the datasets used. Verification using more diverse cases is necessary, and considering the characteristics of rare cancers, data collection through multi-institutional collaborative research is desirable. To enhance data diversity and mitigate overfitting, we employed augmentation techniques such as intensity shifts, affine transformations, and patch-based cropping during model training. These methods were specifically designed to preserve the subtle radiographic shadows characteristic of bone tumors in X-ray images, which are critical for accurate detection. The effectiveness of these techniques was validated through the model’s performance on an independent external dataset, demonstrating their ability to improve robustness and generalization. While advanced techniques such as generative adversarial network (GAN)-based synthetic data generation hold promise for further expanding dataset diversity, their application to high-resolution X-ray images with subtle shadow details remains challenging, particularly in the context of rare diseases like malignant bone tumors. This is primarily due to the limited availability of training data for such diseases, which makes it difficult to develop high-performance GAN models capable of accurately capturing and replicating the fine-grained features necessary for clinical applications [32]. Our proposed 3C method, with its anatomically informed structured annotations, could potentially serve as a foundational approach for developing more sophisticated data augmentation techniques, including GANs specifically tailored for rare diseases. The anatomical separation of tumor regions in our method provides a framework that could guide the generation of more realistic and clinically relevant synthetic data in future research. As GAN architectures and domain-specific adaptations continue to evolve, further investigation will explore how our 3C annotation method can contribute to the development of more effective data augmentation strategies for rare diseases. Additionally, further investigation is needed regarding the optimal methods for three-class annotation and the weighting of each class. Additionally, the ethical challenges associated with AI applications in medicine must be carefully considered. It is crucial for healthcare professionals, researchers, and ethics experts to collaborate to develop guidelines and appropriate educational programs that address various issues, including privacy protection, algorithmic bias, and the interpretation and responsibility of AI diagnoses [33]. The primary focus of this study was to evaluate the effectiveness of the proposed three-class annotation method for improving AI detection of early-stage osteosarcoma. To achieve this, we intentionally employed the standard U-Net architecture without incorporating variations or additional enhancements. This approach allowed us to isolate and assess the impact of the annotation strategy independently of other architectural factors. However, we recognize that a thorough comparison with different U-Net variations and alternative deep learning models would provide a more comprehensive understanding of the proposed method’s robustness and generalizability. Examples of such variations include attention mechanisms, residual connections, or transfer learning approaches, all of which have demonstrated potential in enhancing segmentation and classification tasks in medical imaging. Evaluating the three-class annotation method using these architectures would allow us to better determine its adaptability and potential benefits in diverse clinical scenarios. As part of future work, we plan to conduct additional experiments to compare the performance of the three-class annotation approach across multiple U-Net variations and other architectures. These comparisons will not only validate our findings but also help optimize the annotation strategy for broader applications. This limitation has been added to acknowledge the scope of the current study and to provide transparency about its boundaries. Despite these limitations, we believe that the results of this study provide a significant contribution to the field by demonstrating the potential of anatomically informed annotation strategies in rare cancer diagnosis.

### 4.5. Ethical Considerations and Implementation

The AI model using the three-class annotation method developed in this study introduces new possibilities as a diagnostic approach for primary malignant bone tumors. Particularly, for early-stage abnormality detection and detailed lesion evaluation, it demonstrated characteristics distinct from conventional single-class models, potentially contributing to improved diagnostic accuracy and early detection rates depending on its clinical implementation. Future expectations include verification using larger and more diverse datasets, application to other modalities, and addressing the ethical challenges to further enhance the effectiveness and safety of this new approach.

### 4.6. Global Impact and Future Perspectives

Furthermore, the distinct characteristics revealed between the single- and three-class models demonstrate the importance of matching model characteristics with clinical needs beyond basic performance metrics in AI model development. The successful development of an AI model for primary malignant bone tumors indicates the potential for AI technology applications in other rare diseases, contributing to the advancement of personalized medicine. Furthermore, the three-class annotation method presents an approach for integrating diverse imaging findings within a single disease, a concept that is applicable to other imaging modalities and disease areas. This study emphasized the significance of annotation design for future medical AI development by demonstrating how annotation methods can significantly influence AI model performance and characteristics. Future challenges include verification using larger and more diverse datasets, application to other imaging modalities, and optimization of the three-class annotation method. Moreover, developing explainable AI to visualize AI decision-making and address the ethical and legal issues associated with the clinical implementation of AI diagnostic support tools is crucial. Building on these research outcomes, establishing international research collaboration networks and sharing insights into rare cancer diagnosis and treatment are expected to contribute to improving healthcare quality on a global scale. This study presents a novel approach for improving the diagnostic accuracy and early detection rates of rare cancers by combining AI technology and medical insights. This innovative method is expected to serve as a guideline for future medical imaging diagnostic AI development and contribute to improving the quality of life of patients worldwide. Collaboration among healthcare professionals, AI researchers, and policymakers in promoting further development and appropriate clinical application of this new technology represents a significant step toward realizing next-generation precision medicine.

## 5. Conclusions

The AI model for X-ray image interpretation using the three-class annotation method developed in this study presents an innovative approach for diagnosing primary malignant bone tumors and has significant implications for future medical image diagnostic AI development. By overcoming the limitations of conventional single-class annotation methods and enabling detailed classification of different regions within tumors, this approach has demonstrated the potential to contribute to the early detection of rare cancers and appropriate treatment selection through enhanced AI model learning capabilities and diagnostic characteristics. In screening tests for rare diseases with low prevalence, high specificity is particularly crucial because false positives can lead to excessively detailed examinations. The 3C model demonstrated significant clinical value through its flexibility in detecting subtle abnormalities at lower cutoff values (0.661) while maintaining abnormal region capture capability (DICE coefficient 0.644). This three-class annotation method presents a novel modeling approach for complex diseases with regions of different characteristics within a single lesion, suggesting potential applications in AI diagnostic model development for other rare cancers and diseases with complex lesions.

## Data Availability

The numeric data presented in Figure 4 are available in Table S1 in the Supplementary Materials. Additional data that support the findings of this study are available from the corresponding author upon reasonable request.

## Supplementary Materials

The following supporting information can be downloaded at: https://www.mdpi.com/article/10.3390/cancers17010029/s1, Table S1: Score values and DICE coefficients data for 1C and 3C models shown in Figure 4.

## References

[1] Savage S.A., Mirabello L. (2011). Using Epidemiology and Genomics to Understand Osteosarcoma Etiology. Sarcoma.

[2] Stiller C.A., Bielack S.S., Jundt G., Steliarova-Foucher E. (2006). Bone Tumours in European Children and Adolescents, 1978–1997. Report from the Automated Childhood Cancer Information System Project. Eur. J. Cancer.

[3] Mirabello L., Troisi R.J., Savage S.A. (2009). International Osteosarcoma Incidence Patterns in Children and Adolescents, Middle Ages and Elderly Persons. Int. J. Cancer.

[4] Gardner M.H., Barnes M.J., Bopanna S., Davis C.S., Cotton P.B., Heron B.L., Henninger A., Alva E., Gleason M.W., Whelan K.F. (2014). Barriers to the Use of Psychosocial Support Services Among Adolescent and Young Adult Survivors of Pediatric Cancer. J. Adolesc. Young Adult Oncol..

[5] Bleyer A., Barr R., Ries L., Whelan J., Ferrari A. (2016). Cancer in Adolescents and Young Adults.

[6] Zebrack B.J., Block R., Hayes-Lattin B., Embry L., Aguilar C., Meeske K.A., Li Y., Butler M., Cole S. (2013). Psychosocial Service Use and Unmet Need among Recently Diagnosed Adolescent and Young Adult Cancer Patients. Cancer.

[7] Longhi A., Errani C., De Paolis M., Mercuri M., Bacci G. (2006). Primary Bone Osteosarcoma in the Pediatric Age: State of the Art. Cancer Treat. Rev..

[8] Johnston K.J., Wen H., Joynt Maddox K.E. (2019). Lack of Access to Specialists Associated with Mortality and Preventable Hospitalizations of Rural Medicare Beneficiaries. Health Aff..

[9] Resnick M.J. (2020). Re: Lack of Access to Specialists Associated with Mortality and Preventable Hospitalizations of Rural Medicare Beneficiaries. J. Urol..

[10] Grineski S., Morales D.X., Collins T., Wilkes J., Bonkowsky J.L. (2020). Geographic and Specialty Access Disparities in US Pediatric Leukodystrophy Diagnosis. J. Pediatr..

[11] Obuchowicz R., Strzelecki M., Piórkowski A. (2024). Clinical Applications of Artificial Intelligence in Medical Imaging and Image Processing-A Review. Cancers.

[12] Li M., Jiang Y., Zhang Y., Zhu H. (2023). Medical Image Analysis Using Deep Learning Algorithms. Front. Public Health.

[13] Sistaninejhad B., Rasi H., Nayeri P. (2023). A Review Paper about Deep Learning for Medical Image Analysis. Comput. Math. Methods Med..

[14] Litjens G., Kooi T., Bejnordi B.E., Setio A.A.A., Ciompi F., Ghafoorian M., van der Laak J.A.W.M., van Ginneken B., Sánchez C.I. (2017). A Survey on Deep Learning in Medical Image Analysis. Med. Image Anal..

[15] He Y., Pan I., Bao B., Halsey K., Chang M., Liu H., Peng S., Sebro R.A., Guan J., Yi T. (2020). Deep Learning-Based Classification of Primary Bone Tumors on Radiographs: A Preliminary Study. EBioMedicine.

[16] von Schacky C.E., Wilhelm N.J., Schäfer V.S., Leonhardt Y., Gassert F.G., Foreman S.C., Gassert F.T., Jung M., Jungmann P.M., Russe M.F. (2021). Multitask Deep Learning for Segmentation and Classification of Primary Bone Tumors on Radiographs. Radiology.

[17] Li J., Li S., Li X., Miao S., Dong C., Gao C., Liu X., Hao D., Xu W., Huang M. (2023). Primary Bone Tumor Detection and Classification in Full-Field Bone Radiographs via YOLO Deep Learning Model. Eur. Radiol..

[18] Ouyang T., Yang S., Gou F., Dai Z., Wu J. (2022). Rethinking U-Net from an Attention Perspective with Transformers for Osteosarcoma MRI Image Segmentation. Comput. Intell. Neurosci..

[19] Wu J., Liu Z., Gou F., Zhu J., Tang H., Zhou X., Xiong W. (2022). BA-GCA Net: Boundary-Aware Grid Contextual Attention Net in Osteosarcoma MRI Image Segmentation. Comput. Intell. Neurosci..

[20] Wu J., Zhou L., Gou F., Tan Y. (2022). A Residual Fusion Network for Osteosarcoma MRI Image Segmentation in Developing Countries. Comput. Intell. Neurosci..

[21] Hasei J., Nakahara R., Otsuka Y., Nakamura Y., Hironari T., Kahara N., Miwa S., Ohshika S., Nishimura S., Ikuta K. (2024). High-Quality Expert Annotations Enhance Artificial Intelligence Model Accuracy for Osteosarcoma X-Ray Diagnosis. Cancer Sci..

[22] McDonald J., DenOtter T.D. (2023). Codman Triangle. StatPearls.

[23] Ronneberger O., Fischer P., Brox T. (2015). U-Net: Convolutional Networks for Biomedical Image Segmentation. Proceedings of the Medical Image Computing and Computer-Assisted Intervention—MICCAI 2015.

[24] He K., Zhang X., Ren S., Sun J. Deep Residual Learning for Image Recognition. Proceedings of the 2016 IEEE Conference on Computer Vision and Pattern Recognition (CVPR).

[25] Chlap P., Finnegan R.N. (2023). PlatiPy: Processing Library and Analysis Toolkit for Medical Imaging in Python. J. Open Source Softw..

[26] Smith L.N., Topin N. (2019). Super-Convergence: Very Fast Training of Neural Networks Using Large Learning Rates. Proceedings of the Artificial Intelligence and Machine Learning for Multi-Domain Operations Applications.

[27] Carass A., Roy S., Gherman A., Reinhold J.C., Jesson A., Arbel T., Maier O., Handels H., Ghafoorian M., Platel B. (2020). Evaluating White Matter Lesion Segmentations with Refined Sørensen-Dice Analysis. Sci. Rep..

[28] Fluss R., Faraggi D., Reiser B. (2005). Estimation of the Youden Index and Its Associated Cutoff Point. Biom. J..

[29] Dhamnetiya D., Jha R.P., Shalini S., Bhattacharyya K. (2022). How to Analyze the Diagnostic Performance of a New Test? Explained with Illustrations. J. Lab. Physicians.

[30] Mcnemar Q. (1947). Note on the Sampling Error of the Difference between Correlated Proportions or Percentages. Psychometrika.

[31] Wilson E.B. (1927). Probable Inference, the Law of Succession, and Statistical Inference. J. Am. Stat. Assoc..

[32] Islam S., Aziz M.T., Nabil H.R., Jim J.R., Mridha M.F., Kabir M.M., Asai N., Shin J. (2024). Generative Adversarial Networks (GANs) in Medical Imaging: Advancements, Applications, and Challenges. IEEE Access.

[33] Topol E.J. (2019). High-Performance Medicine: The Convergence of Human and Artificial Intelligence. Nat. Med..

