# Supplementary material for: The Three-Class Annotation Method Improves the AI Detection of Early-Stage Osteosarcoma on Plain Radiographs: A Novel Approach for Rare Cancer Diagnosis
Source: Cancers (Basel). 2024 Dec 25;17(1):29. doi: 10.3390/cancers17010029 (PMC11718825; doi:10.3390/cancers17010029)
Supplement: Supplementary file 1 [file cancers-17-00029-s001.zip › cancers-3345952-supplementary.pdf]

filename	label	1C score	1C Dice	3C score	3C Dice
P:/osteosa	1	0.615921	0.039124	0.860412	0.6235
P:/osteosa	1	0.306789	0	0.799868	0.568474
P:/osteosa	1	0.768783	0	0.469043	0.522599
P:/osteosa	1	0.922565	0.01005	0.934936	0.521463
P:/osteosa	1	0.99997	0.471471	0.725518	0.941378
P:/osteosa	1	0.998557	0.365107	0.677756	0.834535
P:/osteosa	1	0.918028	0.112275	0.89013	0.577647
P:/osteosa	1	0.970331	0.030251	0.704997	0.418976
P:/osteosa	1	0.989803	0.267999	0.922665	0.653111
P:/osteosa	1	0.822034	0.130031	0.724979	0.506507
P:/osteosa	1	0.778409	0.364407	0.141589	0.690309
P:/osteosa	1	0.865193	0.359527	0.989403	0.671119
P:/osteosa	1	0.999162	0.453357	0.995523	0.738034
P:/osteosa	1	0.815996	0.464588	0.317605	0.747768
P:/osteosa	1	0.603075	0.106702	0.298298	0.378087
P:/osteosa	1	0.999889	0.408512	0.75627	0.67087
P:/osteosa	1	0.9936	0.596012	0.994943	0.852116
P:/osteosa	1	0.925521	0.345787	0.903179	0.577847
P:/osteosa	1	0.992244	0.511905	0.955586	0.733681
P:/osteosa	1	0.933957	0.475811	0.697877	0.67823
P:/osteosa	1	0.999845	0.510506	0.858864	0.710706
P:/osteosa	1	0.97192	0.653364	0.999877	0.844574
P:/osteosa	1	0.97462	0.443051	0.365146	0.619034
P:/osteosa	1	0.865663	0.178138	0.927217	0.329315
P:/osteosa	1	0.916441	0.500116	0.534226	0.64304
P:/osteosa	1	0.994129	0.560053	0.524127	0.699605
P:/osteosa	1	0.989861	0.493117	0.84516	0.618632
P:/osteosa	1	0.999705	0.551088	0.758243	0.672266
P:/osteosa	1	0.999945	0.723659	0.646181	0.834137
P:/osteosa	1	0.999876	0.341994	0.978473	0.445642
P:/osteosa	1	0.915389	0.625667	0.795846	0.724188
P:/osteosa	1	0.99902	0.363696	0.950195	0.456622
P:/osteosa	1	0.999706	0.589364	0.903261	0.676549
P:/osteosa	1	0.997339	0.754418	0.555302	0.839497
P:/osteosa	1	0.995447	0.48449	0.700513	0.567429
P:/osteosa	1	0.999133	0.812548	0.810751	0.894936
P:/osteosa	1	0.972289	0.067606	0.874532	0.14949
P:/osteosa	1	0.977513	0.744366	0.990813	0.825788
P:/osteosa	1	0.999935	0.809182	0.953414	0.88946

P:/osteosa	1	0.999511	0.589689	0.435209	0.668116
P:/osteosa	1	0.998753	0.503086	0.986986	0.581051
P:/osteosa	1	0.988342	0.620558	0.814964	0.695709
P:/osteosa	1	0.983198	0.637367	0.805955	0.710424
P:/osteosa	1	0.9999	0.774259	0.689092	0.847128
P:/osteosa	1	0.999712	0.717903	0.998519	0.79004
P:/osteosa	1	0.999838	0.793444	0.997111	0.857937
P:/osteosa	1	0.999503	0.730086	0.999472	0.792593
P:/osteosa	1	0.99997	0.783985	0.949224	0.841109
P:/osteosa	1	0.999729	0.803669	0.670261	0.858634
P:/osteosa	1	0.998983	0.710899	0.967501	0.765516
P:/osteosa	1	0.998549	0.7981	0.999729	0.851561
P:/osteosa	1	0.995935	0.591585	0.701281	0.64379
P:/osteosa	1	0.999105	0.872981	0.487754	0.925142
P:/osteosa	1	0.999962	0.756927	0.764252	0.801559
P:/osteosa	1	0.994911	0.69533	0.976984	0.737567
P:/osteosa	1	0.998378	0.741993	0.899366	0.783266
P:/osteosa	1	0.999948	0.860033	0.734142	0.901196
P:/osteosa	1	0.939258	0.136461	0.396379	0.176897
P:/osteosa	1	0.999155	0.650108	0.537906	0.688746
P:/osteosa	1	0.99983	0.846911	0.998749	0.88297
P:/osteosa	1	0.99954	0.634033	0.985026	0.669581
P:/osteosa	1	0.999963	0.810472	0.95352	0.844697
P:/osteosa	1	0.984622	0.599358	0.644833	0.631202
P:/osteosa	1	0.999955	0.875328	0.999936	0.906918
P:/osteosa	1	0.996954	0.695757	0.988668	0.724274
P:/osteosa	1	0.999874	0.845462	0.391111	0.873779
P:/osteosa	1	0.999862	0.829977	0.886836	0.856872
P:/osteosa	1	0.999739	0.703638	0.997962	0.729021
P:/osteosa	1	0.999595	0.739564	0.999084	0.76479
P:/osteosa	1	0.999947	0.868909	0.999999	0.893956
P:/osteosa	1	0.99949	0.87753	0.951246	0.902288
P:/osteosa	1	0.999847	0.771378	0.365355	0.795796
P:/osteosa	1	0.998544	0.733744	0.513107	0.757921
P:/osteosa	1	0.996155	0.836926	0.849336	0.859719
P:/osteosa	1	0.993494	0.77749	0.162747	0.799264
P:/osteosa	1	0.132149	0	0.47924	0.02139
P:/osteosa	1	0.993601	0.757731	0.967971	0.778909
P:/osteosa	1	0.998406	0.702759	0.905979	0.723558
P:/osteosa	1	0.999891	0.866707	0.946761	0.887356

P:/osteosa	1	0.983163	0.677273	0.974744	0.697622
P:/osteosa	1	0.955629	0.684211	0.817305	0.704265
P:/osteosa	1	0.999925	0.792562	0.607656	0.812526
P:/osteosa	1	0.999744	0.874842	0.687876	0.894665
P:/osteosa	1	0.999971	0.868831	0.950981	0.887701
P:/osteosa	1	0.999315	0.794683	0.99058	0.812957
P:/osteosa	1	0.999764	0.896067	0.999453	0.913365
P:/osteosa	1	0.999802	0.874662	0.997567	0.891068
P:/osteosa	1	0.948576	0.719266	0.984953	0.735556
P:/osteosa	1	0.999921	0.901248	0.999424	0.917197
P:/osteosa	1	0.999672	0.798871	0.895318	0.814292
P:/osteosa	1	0.999976	0.871304	0.99997	0.885313
P:/osteosa	1	0.999883	0.829379	0.545931	0.843387
P:/osteosa	1	0.998867	0.862115	0.705442	0.875009
P:/osteosa	1	0.999894	0.601118	0.988911	0.613195
P:/osteosa	1	0.999835	0.794974	0.999638	0.806977
P:/osteosa	1	0.999745	0.89171	0.999985	0.903709
P:/osteosa	1	0.999966	0.900513	0.999995	0.91233
P:/osteosa	1	0.999851	0.64493	0.743606	0.656138
P:/osteosa	1	0.9993	0.875901	0.999234	0.886514
P:/osteosa	1	0.99792	0.744018	0.953725	0.753407
P:/osteosa	1	0.999446	0.828747	0.720418	0.837762
P:/osteosa	1	0.993918	0.694651	0.98205	0.703101
P:/osteosa	1	0.999706	0.885595	0.95916	0.892961
P:/osteosa	1	0.989999	0.504615	0.868061	0.511884
P:/osteosa	1	0.99992	0.883145	0.991193	0.889553
P:/osteosa	1	0.999315	0.832236	0.984963	0.838131
P:/osteosa	1	0.784851	0.388123	0.13751	0.393843
P:/osteosa	1	0.999981	0.885395	0.955637	0.891001
P:/osteosa	1	0.999173	0.913575	0.995994	0.918314
P:/osteosa	1	0.99986	0.875039	0.998829	0.878287
P:/osteosa	1	0.998071	0.852278	0.995585	0.853713
P:/osteosa	1	0.994489	0.850464	0.997742	0.851679
P:/osteosa	1	0.998201	0.68417	0.959846	0.68533
P:/osteosa	1	0.998995	0.909755	0.822379	0.910351
P:/osteosa	1	0.472583	0	0.147414	0
P:/osteosa	1	0.408539	0	0.06315	0
P:/osteosa	1	0.293736	0	0.066955	0
P:/osteosa	1	0.060717	0	0.020047	0
P:/osteosa	1	0.398147	0	0.131196	0

P:/osteosa	1	0.111737	0	0.046629	0
P:/osteosa	0	0.931116	0	0.70446	0
P:/osteosa	0	0.092433	0	0.024079	0
P:/osteosa	0	0.014794	0	0.117377	0
P:/osteosa	0	0.007819	0	0.032078	0
P:/osteosa	0	0.002884	0	0.003128	0
P:/osteosa	0	0.007339	0	0.016338	0
P:/osteosa	0	0.094945	0	0.015142	0
P:/osteosa	0	0.118699	0	0.009901	0
P:/osteosa	0	0.022453	0	0.01027	0
P:/osteosa	0	0.058586	0	0.002233	0
P:/osteosa	0	0.24773	0	0.139711	0
P:/osteosa	0	0.011353	0	0.025073	0
P:/osteosa	0	0.0084	0	0.010333	0
P:/osteosa	0	0.014199	0	0.003278	0
P:/osteosa	0	0.015091	0	0.008899	0
P:/osteosa	0	0.118032	0	0.015367	0
P:/osteosa	0	0.022263	0	0.034899	0
P:/osteosa	0	0.074807	0	0.065551	0
P:/osteosa	0	0.039734	0	0.170309	0
P:/osteosa	0	0.090582	0	0.007118	0
P:/osteosa	0	0.997838	0	0.263482	0
P:/osteosa	0	0.48695	0	0.645572	0
P:/osteosa	0	0.029772	0	0.05878	0
P:/osteosa	0	0.003253	0	0.007958	0
P:/osteosa	0	0.002967	0	0.05466	0
P:/osteosa	0	0.02622	0	0.005612	0
P:/osteosa	0	0.003969	0	0.003893	0
P:/osteosa	0	0.999126	0	0.948292	0
P:/osteosa	0	0.561277	0	0.973228	0
P:/osteosa	0	0.020225	0	0.008644	0
P:/osteosa	0	0.007214	0	0.004375	0
P:/osteosa	0	0.047688	0	0.014293	0
P:/osteosa	0	0.02143	0	0.025249	0
P:/osteosa	0	0.024383	0	0.00797	0
P:/osteosa	0	0.206739	0	0.068092	0
P:/osteosa	0	0.22761	0	0.318888	0
P:/osteosa	0	0.004855	0	0.014146	0
P:/osteosa	0	0.120833	0	0.600761	0
P:/osteosa	0	0.081556	0	0.04907	0

P:/osteosa	0	0.010635	0	0.003624	0
P:/osteosa	0	0.007102	0	0.008615	0
P:/osteosa	0	0.001804	0	0.002186	0
P:/osteosa	0	0.002217	0	0.022107	0
P:/osteosa	0	0.036266	0	0.004625	0
P:/osteosa	0	0.048093	0	0.03717	0
P:/osteosa	0	0.664218	0	0.717998	0
P:/osteosa	0	0.098602	0	0.043194	0
P:/osteosa	0	0.057419	0	0.008467	0
P:/osteosa	0	0.017806	0	0.012921	0
P:/osteosa	0	0.003892	0	0.015015	0
P:/osteosa	0	0.031506	0	0.011696	0
P:/osteosa	0	0.281505	0	0.042661	0
P:/osteosa	0	0.003949	0	0.024641	0
P:/osteosa	0	0.005442	0	0.013114	0
P:/osteosa	0	0.042288	0	0.038609	0
P:/osteosa	0	0.024006	0	0.107933	0
P:/osteosa	0	0.325943	0	0.063651	0
P:/osteosa	0	0.837627	0	0.249135	0
P:/osteosa	0	0.75621	0	0.729805	0
P:/osteosa	0	0.009972	0	0.006576	0
P:/osteosa	0	0.055263	0	0.004402	0
P:/osteosa	0	0.053005	0	0.130611	0
P:/osteosa	0	0.092512	0	0.020981	0
P:/osteosa	0	0.388448	0	0.66794	0
P:/osteosa	0	0.796998	0	0.088173	0
P:/osteosa	0	0.274512	0	0.276225	0
P:/osteosa	0	0.002918	0	0.000988	0
P:/osteosa	0	0.04962	0	0.015984	0
P:/osteosa	0	0.015147	0	0.103915	0
P:/osteosa	0	0.025168	0	0.004045	0
P:/osteosa	0	0.007035	0	0.006568	0
P:/osteosa	0	0.042176	0	0.009403	0
P:/osteosa	0	0.174844	0	0.015896	0
P:/osteosa	0	0.006608	0	0.002872	0
P:/osteosa	0	0.029529	0	0.034478	0
P:/osteosa	0	0.332962	0	0.188678	0
P:/osteosa	0	0.043261	0	0.003499	0
P:/osteosa	0	0.0095	0	0.013339	0
P:/osteosa	0	0.329207	0	0.079919	0

P:/osteosa	0	0.461076	0	0.042623	0
P:/osteosa	0	0.034919	0	0.021079	0
P:/osteosa	0	0.163677	0	0.049134	0
P:/osteosa	0	0.578638	0	0.759541	0
P:/osteosa	0	0.018136	0	0.001943	0
P:/osteosa	0	0.217495	0	0.008619	0
P:/osteosa	0	0.014451	0	0.003061	0
P:/osteosa	0	0.033697	0	0.078487	0
P:/osteosa	0	0.020056	0	0.008087	0
P:/osteosa	0	0.067914	0	0.003917	0
P:/osteosa	0	0.050886	0	0.029282	0
P:/osteosa	0	0.296822	0	0.073295	0
P:/osteosa	0	0.017038	0	0.023237	0
P:/osteosa	0	0.057919	0	0.010384	0
P:/osteosa	0	0.429002	0	0.049328	0
P:/osteosa	0	0.216239	0	0.012281	0
P:/osteosa	0	0.027534	0	0.016343	0
P:/osteosa	0	0.004863	0	0.029276	0
P:/osteosa	0	0.016367	0	0.012404	0
P:/osteosa	0	0.310321	0	0.395693	0
P:/osteosa	0	0.090667	0	0.088907	0
P:/osteosa	0	0.010237	0	0.056045	0
P:/osteosa	0	0.067951	0	0.22609	0
P:/osteosa	0	0.126648	0	0.011166	0
P:/osteosa	0	0.027527	0	0.108365	0
P:/osteosa	0	0.090077	0	0.006963	0
P:/osteosa	0	0.04768	0	0.012963	0
P:/osteosa	0	0.003404	0	0.008909	0
P:/osteosa	0	0.016157	0	0.028395	0
P:/osteosa	0	0.031645	0	0.02424	0
P:/osteosa	0	0.274798	0	0.040281	0
P:/osteosa	0	0.014938	0	0.00318	0
P:/osteosa	0	0.547724	0	0.092548	0
P:/osteosa	0	0.098912	0	0.078082	0
P:/osteosa	0	0.985034	0	0.741639	0
P:/osteosa	0	0.031371	0	0.035814	0
P:/osteosa	0	0.074852	0	0.027464	0
P:/osteosa	0	0.036489	0	0.03313	0
P:/osteosa	0	0.966635	0	0.942782	0
P:/osteosa	0	0.003659	0	0.006234	0

P:/osteosa	0	0.02226	0	0.016506	0
P:/osteosa	0	0.070552	0	0.022776	0
P:/osteosa	0	0.011843	0	0.056496	0
P:/osteosa	0	0.06807	0	0.044375	0
P:/osteosa	0	0.001284	0	0.000792	0
P:/osteosa	0	0.024846	0	0.00526	0
P:/osteosa	0	0.126639	0	0.021583	0
P:/osteosa	0	0.01838	0	0.010588	0
P:/osteosa	0	0.081246	0	0.013045	0
P:/osteosa	0	0.026431	0	0.005071	0
P:/osteosa	0	0.007989	0	0.057695	0
P:/osteosa	0	0.011307	0	0.019942	0
P:/osteosa	0	0.039518	0	0.021501	0
P:/osteosa	0	0.021974	0	0.007102	0
P:/osteosa	0	0.097738	0	0.009041	0
P:/osteosa	0	0.055011	0	0.353202	0
P:/osteosa	0	0.492214	0	0.011376	0
P:/osteosa	0	0.008751	0	0.006946	0
P:/osteosa	0	0.036775	0	0.001638	0
P:/osteosa	0	0.792291	0	0.84849	0
P:/osteosa	0	0.335543	0	0.009696	0
P:/osteosa	0	0.099872	0	0.110588	0
P:/osteosa	0	0.003095	0	0.003673	0
P:/osteosa	0	0.016841	0	0.010906	0
P:/osteosa	0	0.009078	0	0.015577	0
P:/osteosa	0	0.027106	0	0.003186	0
P:/osteosa	0	0.028205	0	0.028576	0
P:/osteosa	0	0.146089	0	0.013724	0
P:/osteosa	0	0.071627	0	0.032427	0
P:/osteosa	0	0.018754	0	0.16256	0
P:/osteosa	0	0.041127	0	0.0688	0
P:/osteosa	0	0.003814	0	0.020247	0
P:/osteosa	0	0.02858	0	0.039869	0
P:/osteosa	0	0.018485	0	0.485406	0
P:/osteosa	0	0.176187	0	0.017743	0
P:/osteosa	0	0.093734	0	0.037302	0
P:/osteosa	0	0.005687	0	0.045454	0
P:/osteosa	0	0.117902	0	0.006323	0
P:/osteosa	0	0.026266	0	0.021268	0
P:/osteosa	0	0.050065	0	0.002698	0

P:/osteosa	0	0.022579	0	0.012397	0
P:/osteosa	0	0.588141	0	0.154459	0
P:/osteosa	0	0.026482	0	0.013815	0
P:/osteosa	0	0.122765	0	0.046518	0
P:/osteosa	0	0.003883	0	0.009466	0
P:/osteosa	0	0.035051	0	0.035295	0
P:/osteosa	0	0.14382	0	0.021493	0
P:/osteosa	0	0.164777	0	0.020397	0
P:/osteosa	0	0.070568	0	0.044529	0
P:/osteosa	0	0.039581	0	0.013203	0
P:/osteosa	0	0.031042	0	0.037054	0
P:/osteosa	0	0.017136	0	0.002414	0
P:/osteosa	0	0.02303	0	0.003123	0
P:/osteosa	0	0.066437	0	0.026986	0
P:/osteosa	0	0.030657	0	0.021736	0
P:/osteosa	0	0.058473	0	0.053433	0
P:/osteosa	0	0.013983	0	0.003582	0
P:/osteosa	0	0.009639	0	0.021484	0
P:/osteosa	0	0.006244	0	0.012892	0
P:/osteosa	0	0.994025	0	0.175268	0
P:/osteosa	0	0.967054	0	0.848713	0
P:/osteosa	0	0.055694	0	0.006729	0
P:/osteosa	0	0.477838	0	0.129212	0
P:/osteosa	0	0.13219	0	0.00524	0
P:/osteosa	0	0.024891	0	0.013758	0
P:/osteosa	0	0.009498	0	0.070249	0
P:/osteosa	0	0.388794	0	0.035879	0
P:/osteosa	0	0.010759	0	0.003962	0
P:/osteosa	0	0.007052	0	0.023909	0
P:/osteosa	0	0.040001	0	0.028758	0
P:/osteosa	0	0.425075	0	0.047105	0
P:/osteosa	0	0.004926	0	0.062077	0
P:/osteosa	0	0.014328	0	0.026916	0
P:/osteosa	0	0.022009	0	0.01224	0
P:/osteosa	0	0.015835	0	0.030122	0
P:/osteosa	0	0.006854	0	0.003775	0
P:/osteosa	0	0.037624	0	0.00937	0
P:/osteosa	0	0.040124	0	0.014573	0
P:/osteosa	0	0.02023	0	0.006	0
P:/osteosa	0	0.385143	0	0.016311	0

P:/osteosa	0	0.030718	0	0.019311	0
P:/osteosa	0	0.015134	0	0.023036	0
P:/osteosa	0	0.05271	0	0.048598	0
P:/osteosa	0	0.001543	0	0.001433	0
P:/osteosa	0	0.017829	0	0.008145	0
P:/osteosa	0	0.014203	0	0.006485	0
P:/osteosa	0	0.008391	0	0.006045	0
P:/osteosa	0	0.305472	0	0.113218	0
P:/osteosa	0	0.012447	0	0.003776	0
P:/osteosa	0	0.008452	0	0.01411	0
P:/osteosa	0	0.005304	0	0.025289	0
P:/osteosa	0	0.02339	0	0.002949	0
P:/osteosa	0	0.005805	0	0.002833	0
P:/osteosa	0	0.917999	0	0.055582	0
P:/osteosa	0	0.041722	0	0.071533	0
P:/osteosa	0	0.130938	0	0.123871	0
P:/osteosa	0	0.010438	0	0.008878	0
P:/osteosa	0	0.053174	0	0.048511	0
P:/osteosa	0	0.068991	0	0.005504	0
P:/osteosa	0	0.01627	0	0.045097	0
P:/osteosa	0	0.04666	0	0.014243	0
P:/osteosa	0	0.005763	0	0.011141	0
P:/osteosa	0	0.014808	0	0.008035	0
P:/osteosa	0	0.006718	0	0.011218	0
P:/osteosa	0	0.00294	0	0.004503	0
P:/osteosa	0	0.01855	0	0.002581	0
P:/osteosa	0	0.037888	0	0.045692	0
P:/osteosa	0	0.381663	0	0.011404	0
P:/osteosa	0	0.011782	0	0.037744	0
P:/osteosa	0	0.13223	0	0.058356	0
P:/osteosa	0	0.011546	0	0.069837	0
P:/osteosa	0	0.022173	0	0.027677	0
P:/osteosa	0	0.241	0	0.090466	0
P:/osteosa	0	0.220345	0	0.076862	0
P:/osteosa	0	0.623997	0	0.451468	0
P:/osteosa	0	0.038594	0	0.110771	0
P:/osteosa	0	0.065537	0	0.010554	0
P:/osteosa	0	0.035237	0	0.011073	0
P:/osteosa	0	0.068539	0	0.006758	0
P:/osteosa	0	0.004197	0	0.00176	0

P:/osteosa	0	0.032455	0	0.049755	0
P:/osteosa	0	0.017149	0	0.017277	0
P:/osteosa	0	0.002403	0	0.011074	0
P:/osteosa	0	0.024463	0	0.012052	0
P:/osteosa	0	0.760761	0	0.264479	0
P:/osteosa	0	0.374121	0	0.336569	0
P:/osteosa	0	0.826777	0	0.288265	0
P:/osteosa	0	0.066978	0	0.00479	0
P:/osteosa	0	0.004616	0	0.002148	0
P:/osteosa	0	0.009479	0	0.138849	0
P:/osteosa	0	0.013527	0	0.046892	0
P:/osteosa	0	0.02146	0	0.022245	0
P:/osteosa	0	0.166225	0	0.013655	0
P:/osteosa	0	0.026576	0	0.036181	0
P:/osteosa	0	0.004888	0	0.032209	0
P:/osteosa	0	0.00206	0	0.005189	0
P:/osteosa	0	0.014982	0	0.042306	0
P:/osteosa	0	0.028322	0	0.019585	0
P:/osteosa	0	0.016229	0	0.031885	0
P:/osteosa	0	0.030523	0	0.020206	0
P:/osteosa	0	0.035262	0	0.007377	0
P:/osteosa	0	0.02499	0	0.005969	0
P:/osteosa	0	0.0502	0	0.010822	0
P:/osteosa	0	0.55009	0	0.087094	0
P:/osteosa	0	0.355889	0	0.067583	0
P:/osteosa	0	0.136989	0	0.052142	0
P:/osteosa	0	0.105728	0	0.024132	0
P:/osteosa	0	0.0694	0	0.005733	0
P:/osteosa	0	0.028614	0	0.005505	0
P:/osteosa	0	0.058009	0	0.123107	0
P:/osteosa	0	0.126471	0	0.074538	0
P:/osteosa	0	0.166753	0	0.060663	0
P:/osteosa	0	0.014273	0	0.005177	0
P:/osteosa	0	0.345738	0	0.132745	0
P:/osteosa	0	0.012885	0	0.082256	0
P:/osteosa	0	0.341515	0	0.023652	0
P:/osteosa	0	0.016033	0	0.009204	0
P:/osteosa	0	0.034142	0	0.055829	0
P:/osteosa	0	0.113771	0	0.013319	0
P:/osteosa	0	0.591698	0	0.060989	0

P:/osteosa	0	0.030507	0	0.070377	0
P:/osteosa	0	0.020773	0	0.004529	0
P:/osteosa	0	0.076736	0	0.012191	0
P:/osteosa	0	0.178078	0	0.002818	0
P:/osteosa	0	0.581075	0	0.301824	0
P:/osteosa	0	0.054721	0	0.00515	0
P:/osteosa	0	0.11582	0	0.148069	0
P:/osteosa	0	0.055397	0	0.04433	0
P:/osteosa	0	0.094451	0	0.001364	0
P:/osteosa	0	0.031655	0	0.027275	0
P:/osteosa	0	0.032748	0	0.007996	0
P:/osteosa	0	0.222052	0	0.227286	0
P:/osteosa	0	0.004948	0	0.001576	0
P:/osteosa	0	0.017314	0	0.109845	0
P:/osteosa	0	0.020768	0	0.052774	0
P:/osteosa	0	0.022264	0	0.033178	0
P:/osteosa	0	0.004839	0	0.002671	0
P:/osteosa	0	0.018866	0	0.005653	0
P:/osteosa	0	0.003671	0	0.003994	0
P:/osteosa	0	0.088698	0	0.202535	0
P:/osteosa	0	0.046096	0	0.02781	0
P:/osteosa	0	0.027159	0	0.016833	0
P:/osteosa	0	0.01469	0	0.037308	0
P:/osteosa	0	0.024642	0	0.034474	0
P:/osteosa	0	0.015233	0	0.012663	0
P:/osteosa	0	0.003563	0	0.006124	0
P:/osteosa	0	0.018372	0	0.005958	0
P:/osteosa	0	0.001888	0	0.003045	0
P:/osteosa	0	0.030039	0	0.004383	0
P:/osteosa	0	0.013029	0	0.069386	0
P:/osteosa	0	0.025235	0	0.008502	0
P:/osteosa	0	0.047047	0	0.015005	0
P:/osteosa	0	0.014141	0	0.006579	0
P:/osteosa	0	0.01085	0	0.004181	0
P:/osteosa	0	0.057965	0	0.001098	0
P:/osteosa	0	0.468224	0	0.204913	0
P:/osteosa	0	0.019076	0	0.010076	0
P:/osteosa	0	0.406394	0	0.010067	0
P:/osteosa	0	0.01456	0	0.02229	0
P:/osteosa	0	0.004686	0	0.011428	0

P:/osteosa	0	0.031653	0	0.007505	0
P:/osteosa	0	0.077919	0	0.013205	0
P:/osteosa	0	0.08445	0	0.016935	0
P:/osteosa	0	0.021031	0	0.001704	0
P:/osteosa	0	0.11701	0	0.014296	0
P:/osteosa	0	0.008099	0	0.003197	0
P:/osteosa	0	0.474615	0	0.048202	0
P:/osteosa	0	0.010892	0	0.022891	0
P:/osteosa	0	0.004158	0	0.004626	0
P:/osteosa	0	0.094943	0	0.004104	0
P:/osteosa	0	0.152265	0	0.019103	0
P:/osteosa	0	0.103259	0	0.024271	0
P:/osteosa	0	0.022432	0	0.036858	0
P:/osteosa	0	0.011903	0	0.001476	0
P:/osteosa	0	0.021866	0	0.004028	0
P:/osteosa	0	0.007776	0	0.003453	0
P:/osteosa	0	0.021981	0	0.005891	0
P:/osteosa	0	0.00197	0	0.004389	0
P:/osteosa	0	0.014957	0	0.020237	0
P:/osteosa	0	0.010088	0	0.008013	0
P:/osteosa	0	0.010348	0	0.010559	0
P:/osteosa	0	0.683113	0	0.004229	0
P:/osteosa	0	0.011704	0	0.104018	0
P:/osteosa	0	0.202119	0	0.052548	0
P:/osteosa	0	0.098985	0	0.012562	0
P:/osteosa	0	0.047625	0	0.002921	0
P:/osteosa	0	0.053352	0	0.068813	0
P:/osteosa	0	0.012628	0	0.008531	0
P:/osteosa	0	0.01715	0	0.005921	0
P:/osteosa	0	0.00937	0	0.004691	0
P:/osteosa	0	0.036281	0	0.019274	0
P:/osteosa	0	0.004545	0	0.008858	0
P:/osteosa	0	0.002518	0	0.008188	0
P:/osteosa	0	0.065611	0	0.013978	0
P:/osteosa	0	0.04565	0	0.013123	0
P:/osteosa	0	0.073949	0	0.024854	0
P:/osteosa	0	0.022323	0	0.010831	0
P:/osteosa	0	0.076627	0	0.010956	0
P:/osteosa	0	0.021165	0	0.008833	0
P:/osteosa	0	0.006202	0	0.003484	0

P:/osteosa	0	0.004453	0	0.010875	0
P:/osteosa	0	0.03484	0	0.001771	0
P:/osteosa	0	0.011642	0	0.006901	0
P:/osteosa	0	0.096139	0	0.025462	0
P:/osteosa	0	0.365109	0	0.003582	0
P:/osteosa	0	0.035193	0	0.019196	0
P:/osteosa	0	0.010641	0	0.00291	0
P:/osteosa	0	0.027158	0	0.008819	0
P:/osteosa	0	0.006152	0	0.004131	0
P:/osteosa	0	0.130991	0	0.012965	0
P:/osteosa	0	0.00969	0	0.001934	0
P:/osteosa	0	0.069534	0	0.060291	0
P:/osteosa	0	0.018766	0	0.027374	0
P:/osteosa	0	0.055956	0	0.014042	0
P:/osteosa	0	0.003617	0	0.004357	0
P:/osteosa	0	0.056871	0	0.007534	0
P:/osteosa	0	0.039386	0	0.011443	0
P:/osteosa	0	0.003787	0	0.005849	0
P:/osteosa	0	0.011413	0	0.007173	0
P:/osteosa	0	0.008607	0	0.002308	0
P:/osteosa	0	0.058795	0	0.008122	0
P:/osteosa	0	0.059197	0	0.007727	0
P:/osteosa	0	0.170223	0	0.008448	0
P:/osteosa	0	0.359053	0	0.237989	0
P:/osteosa	0	0.008458	0	0.010155	0
P:/osteosa	0	0.026205	0	0.045361	0
P:/osteosa	0	0.005096	0	0.00272	0
P:/osteosa	0	0.052837	0	0.010854	0
P:/osteosa	0	0.154719	0	0.009339	0
P:/osteosa	0	0.203257	0	0.031547	0
P:/osteosa	0	0.029848	0	0.008761	0
P:/osteosa	0	0.558677	0	0.432875	0
P:/osteosa	0	0.796909	0	0.013441	0
P:/osteosa	0	0.011199	0	0.009094	0
P:/osteosa	0	0.067519	0	0.407721	0
P:/osteosa	0	0.012437	0	0.013698	0
P:/osteosa	0	0.067033	0	0.221705	0
P:/osteosa	0	0.407984	0	0.049862	0
P:/osteosa	0	0.001025	0	0.000997	0
P:/osteosa	0	0.043651	0	0.005689	0

P:/osteosa	0	0.01875	0	0.010587	0
P:/osteosa	0	0.009487	0	0.005398	0
P:/osteosa	0	0.011468	0	0.003049	0
P:/osteosa	0	0.704369	0	0.082947	0
P:/osteosa	0	0.026079	0	0.005741	0
P:/osteosa	0	0.448479	0	0.014179	0
P:/osteosa	0	0.052101	0	0.022024	0
P:/osteosa	0	0.004074	0	0.021128	0
P:/osteosa	0	0.00801	0	0.010007	0
P:/osteosa	0	0.51066	0	0.008451	0
P:/osteosa	0	0.021199	0	0.01749	0
P:/osteosa	0	0.060576	0	0.009656	0
P:/osteosa	0	0.107445	0	0.028883	0
P:/osteosa	0	0.008314	0	0.00599	0
P:/osteosa	0	0.010617	0	0.057551	0
P:/osteosa	0	0.010957	0	0.001718	0
P:/osteosa	0	0.090203	0	0.009099	0
P:/osteosa	0	0.005389	0	0.004023	0
P:/osteosa	0	0.016783	0	0.007729	0
P:/osteosa	0	0.005702	0	0.023278	0
P:/osteosa	0	0.006224	0	0.004361	0
P:/osteosa	0	0.463668	0	0.017142	0
P:/osteosa	0	0.001446	0	0.004897	0
P:/osteosa	0	0.007071	0	0.016052	0
P:/osteosa	0	0.005813	0	0.004612	0
P:/osteosa	0	0.008684	0	0.007822	0
P:/osteosa	0	0.045509	0	0.006087	0
P:/osteosa	0	0.060794	0	0.004166	0
P:/osteosa	0	0.005093	0	0.001474	0
P:/osteosa	0	0.030134	0	0.006806	0
P:/osteosa	0	0.020013	0	0.004304	0
P:/osteosa	0	0.000969	0	0.006741	0
P:/osteosa	0	0.024204	0	0.014566	0
P:/osteosa	0	0.07714	0	0.30012	0
P:/osteosa	0	0.028406	0	0.012387	0
P:/osteosa	0	0.882951	0	0.693758	0
P:/osteosa	0	0.165599	0	0.018164	0
P:/osteosa	0	0.959776	0	0.832896	0
P:/osteosa	0	0.002753	0	0.001285	0
P:/osteosa	0	0.00151	0	0.000574	0

P:/osteosa	0	0.023573	0	0.00869	0
P:/osteosa	0	0.001357	0	0.006829	0
P:/osteosa	0	0.072273	0	0.01499	0
P:/osteosa	0	0.043297	0	0.01055	0
P:/osteosa	0	0.004635	0	0.002048	0
P:/osteosa	0	0.777329	0	0.017062	0
P:/osteosa	0	0.027582	0	0.059723	0
P:/osteosa	0	0.01144	0	0.012513	0
P:/osteosa	0	0.908266	0	0.27401	0
P:/osteosa	0	0.004341	0	0.003723	0
P:/osteosa	0	0.294959	0	0.015974	0
P:/osteosa	0	0.011812	0	0.017576	0
P:/osteosa	0	0.047816	0	0.040846	0
P:/osteosa	0	0.030291	0	0.004497	0
P:/osteosa	0	0.035819	0	0.027612	0
P:/osteosa	0	0.0568	0	0.046868	0
P:/osteosa	0	0.088149	0	0.083516	0
P:/osteosa	0	0.951733	0	0.165037	0
P:/osteosa	0	0.008935	0	0.006471	0
P:/osteosa	0	0.026068	0	0.025877	0
P:/osteosa	0	0.017825	0	0.004844	0
P:/osteosa	0	0.070604	0	0.031311	0
P:/osteosa	0	0.700866	0	0.052932	0
P:/osteosa	0	0.028721	0	0.018757	0
P:/osteosa	0	0.299093	0	0.029465	0
P:/osteosa	0	0.007142	0	0.046941	0
P:/osteosa	0	0.008629	0	0.019189	0
P:/osteosa	0	0.004133	0	0.006193	0
P:/osteosa	0	0.004176	0	0.005978	0
P:/osteosa	0	0.042777	0	0.011821	0
P:/osteosa	0	0.012987	0	0.026299	0
P:/osteosa	0	0.012456	0	0.011818	0
P:/osteosa	0	0.014501	0	0.033739	0
P:/osteosa	0	0.026581	0	0.013654	0
P:/osteosa	0	0.059257	0	0.033407	0
P:/osteosa	0	0.062078	0	0.032229	0
P:/osteosa	1	0.998574	0.81852	0.996697	0.818394
P:/osteosa	1	0.999851	0.900553	0.983369	0.899973
P:/osteosa	1	0.999943	0.854659	0.999693	0.853177
P:/osteosa	1	0.999937	0.8958	0.998847	0.893971

P:/osteosa	1	0.999536	0.481175	0.970806	0.479284
P:/osteosa	1	0.999967	0.820574	0.77275	0.818624
P:/osteosa	1	0.984718	0.573002	0.630407	0.570631
P:/osteosa	1	0.999894	0.895771	0.829152	0.892929
P:/osteosa	1	0.980454	0.70337	0.766182	0.700289
P:/osteosa	1	0.999577	0.71077	0.920074	0.707285
P:/osteosa	1	0.999856	0.880817	0.99945	0.877328
P:/osteosa	1	0.980615	0.713019	0.23377	0.709406
P:/osteosa	1	0.997327	0.804907	0.740597	0.799442
P:/osteosa	1	0.999893	0.845201	0.999967	0.838437
P:/osteosa	1	0.999899	0.866941	0.775399	0.860024
P:/osteosa	1	0.99971	0.756418	0.965649	0.749481
P:/osteosa	1	0.999518	0.80122	0.669279	0.793533
P:/osteosa	1	0.996095	0.787885	0.902339	0.776416
P:/osteosa	1	0.999896	0.83443	0.802688	0.822591
P:/osteosa	1	0.999152	0.920354	0.638175	0.908361
P:/osteosa	1	0.99989	0.905674	0.995831	0.893388
P:/osteosa	1	0.999828	0.694618	0.693246	0.681205
P:/osteosa	1	0.799189	0.013752	0.452132	0
P:/osteosa	1	0.997675	0.857001	0.998598	0.842487
P:/osteosa	1	0.999314	0.753202	0.991174	0.738051
P:/osteosa	1	0.998536	0.863571	0.996771	0.847896
P:/osteosa	1	0.999949	0.929112	0.964532	0.912774
P:/osteosa	1	0.999919	0.890817	0.754376	0.873832
P:/osteosa	1	0.999141	0.767002	0.986608	0.748483
P:/osteosa	1	0.987579	0.71726	0.966306	0.698685
P:/osteosa	1	0.999987	0.769482	0.770552	0.75
P:/osteosa	1	0.999644	0.861621	0.998787	0.839199
P:/osteosa	1	0.999864	0.71681	0.388929	0.693814
P:/osteosa	1	0.999555	0.798874	0.982769	0.774815
P:/osteosa	1	0.999441	0.796117	0.991025	0.771422
P:/osteosa	1	0.999997	0.805264	0.723629	0.780268
P:/osteosa	1	0.999852	0.803761	0.999497	0.778547
P:/osteosa	1	0.864817	0.628766	0.076691	0.601753
P:/osteosa	1	0.999111	0.937292	0.999334	0.909987
P:/osteosa	1	0.99691	0.762657	0.76714	0.73501
P:/osteosa	1	0.835439	0.461568	0.916958	0.433525
P:/osteosa	1	0.999692	0.728026	0.998591	0.698924
P:/osteosa	1	0.997896	0.792817	0.856339	0.762533
P:/osteosa	1	0.999104	0.812865	0.993393	0.782128

P:/osteosa	1	0.92211	0.031439	0.481488	0
P:/osteosa	1	0.646601	0.03281	0.256718	0
P:/osteosa	1	0.999505	0.79726	0.788009	0.762787
P:/osteosa	1	0.999457	0.492611	0.905778	0.457181
P:/osteosa	1	0.999072	0.707746	0.7931	0.671904
P:/osteosa	1	0.999531	0.829742	0.88765	0.792325
P:/osteosa	1	0.993775	0.630978	0.816448	0.592583
P:/osteosa	1	0.999856	0.92264	0.992572	0.883771
P:/osteosa	1	0.999018	0.831137	0.996906	0.791953
P:/osteosa	1	0.99752	0.829905	0.948805	0.79061
P:/osteosa	1	0.996223	0.738217	0.69255	0.69579
P:/osteosa	1	0.999585	0.90895	0.998884	0.866043
P:/osteosa	1	0.999657	0.869086	0.998995	0.82573
P:/osteosa	1	0.999914	0.941842	0.998629	0.897616
P:/osteosa	1	0.999803	0.822748	0.998783	0.777717
P:/osteosa	1	0.997094	0.722545	0.972685	0.67675
P:/osteosa	1	0.997168	0.809399	0.965873	0.763587
P:/osteosa	1	0.999095	0.851259	0.997043	0.803422
P:/osteosa	1	0.999865	0.881299	0.999993	0.8334
P:/osteosa	1	0.844716	0.667111	0.168439	0.618919
P:/osteosa	1	0.999161	0.772114	0.868906	0.722765
P:/osteosa	1	0.995935	0.58842	0.970986	0.538383
P:/osteosa	1	0.994512	0.883162	0.950143	0.832202
P:/osteosa	1	0.996715	0.597957	0.896651	0.544234
P:/osteosa	1	0.960582	0.492161	0.165592	0.43772
P:/osteosa	1	0.999749	0.844559	0.698823	0.788765
P:/osteosa	1	0.999957	0.744988	0.666245	0.686965
P:/osteosa	1	0.999957	0.777563	0.984503	0.719234
P:/osteosa	1	0.996568	0.774874	0.346176	0.713659
P:/osteosa	1	0.999334	0.684645	0.822151	0.62337
P:/osteosa	1	0.995986	0.807863	0.855347	0.744249
P:/osteosa	1	0.985172	0.657608	0.995466	0.591114
P:/osteosa	1	0.997139	0.853197	0.906733	0.785429
P:/osteosa	1	0.997291	0.842952	0.914485	0.775171
P:/osteosa	1	0.997411	0.710607	0.92235	0.640919
P:/osteosa	1	0.999037	0.753269	0.975412	0.682553
P:/osteosa	1	0.998913	0.755829	0.39245	0.680713
P:/osteosa	1	0.79513	0.093264	0.310749	0.018062
P:/osteosa	1	0.999734	0.839892	0.991304	0.764263
P:/osteosa	1	0.972994	0.782842	0.985425	0.706934

P:/osteosa	1	0.995696	0.751537	0.994376	0.672355
P:/osteosa	1	0.988169	0.815395	0.507996	0.734913
P:/osteosa	1	0.985883	0.720327	0.594239	0.638889
P:/osteosa	1	0.999882	0.920548	0.99989	0.837871
P:/osteosa	1	0.999782	0.743225	0.98862	0.655699
P:/osteosa	1	0.999964	0.889999	0.971576	0.800999
P:/osteosa	1	0.580338	0.093573	0.204766	0
P:/osteosa	1	0.999252	0.827093	0.578747	0.73091
P:/osteosa	1	0.999472	0.897313	0.989984	0.798757
P:/osteosa	1	0.994526	0.648003	0.777517	0.544476
P:/osteosa	1	0.998862	0.5664	0.991999	0.461405
P:/osteosa	1	0.996295	0.643228	0.188157	0.537313
P:/osteosa	1	0.999793	0.735002	0.99846	0.628513
P:/osteosa	1	0.999786	0.928484	0.995618	0.821895
P:/osteosa	1	0.997656	0.722999	0.862538	0.612177
P:/osteosa	1	0.998104	0.863942	0.889585	0.751744
P:/osteosa	1	0.962826	0.57923	0.844688	0.466933
P:/osteosa	1	0.986445	0.737142	0.932939	0.62484
P:/osteosa	1	0.997475	0.538345	0.21647	0.425989
P:/osteosa	1	0.995758	0.547748	0.976615	0.430664
P:/osteosa	1	0.997216	0.650164	0.950742	0.530035
P:/osteosa	1	0.909467	0.184044	0.800983	0.060219
P:/osteosa	1	0.992739	0.698915	0.979728	0.572768
P:/osteosa	1	0.997158	0.610684	0.601607	0.482508
P:/osteosa	1	0.986921	0.826578	0.806653	0.698289
P:/osteosa	1	0.999912	0.814139	0.891464	0.683259
P:/osteosa	1	0.954697	0.542422	0.350461	0.408573
P:/osteosa	1	0.99088	0.769939	0.629657	0.6294
P:/osteosa	1	0.99918	0.829432	0.979903	0.687833
P:/osteosa	1	0.979139	0.347769	0.909533	0.202323
P:/osteosa	1	0.998893	0.726968	0.986576	0.580259
P:/osteosa	1	0.999725	0.897568	0.999378	0.745902
P:/osteosa	1	0.929609	0.225264	0.707935	0.067161
P:/osteosa	1	0.998882	0.532142	0.886761	0.368659
P:/osteosa	1	0.959201	0.59269	0.720016	0.426428
P:/osteosa	1	0.999786	0.859325	0.995549	0.690199
P:/osteosa	1	0.874028	0.404848	0.55293	0.234714
P:/osteosa	1	0.925125	0.583685	0.846763	0.406532
P:/osteosa	1	0.921079	0.606585	0.440997	0.429278
P:/osteosa	1	0.999684	0.697229	0.999504	0.518934

P:/osteosa	1	0.999058	0.863234	0.683563	0.676354
P:/osteosa	1	0.999787	0.864463	0.432225	0.669913
P:/osteosa	1	0.999999	0.727057	0.637412	0.511525
P:/osteosa	1	0.884473	0.288714	0.54354	0.047002
P:/osteosa	1	0.999863	0.91356	0.994473	0.666171
P:/osteosa	1	0.999984	0.831853	0.886946	0.581697
P:/osteosa	1	0.989493	0.674338	0.908213	0.422709
P:/osteosa	1	0.990858	0.530167	0.440143	0.260959
P:/osteosa	1	0.989843	0.441834	0.148391	0.157588
P:/osteosa	1	0.952484	0.289303	0.075946	0
P:/osteosa	1	0.98813	0.871084	0.959612	0.561759
P:/osteosa	1	0.934243	0.633408	0.832645	0.298503
P:/osteosa	1	0.816487	0.63954	0.065927	0.269581
P:/osteosa	1	0.944878	0.68108	0.985325	0.294856
P:/osteosa	1	0.992071	0.858885	0.978883	0.469594
P:/osteosa	1	0.98954	0.796948	0.496577	0.394326
P:/osteosa	1	0.984473	0.759403	0.699458	0.345865
P:/osteosa	1	0.998807	0.782016	0.368869	0.368192
P:/osteosa	1	0.989355	0.645358	0.400038	0.228947
P:/osteosa	1	0.841463	0.417714	0.111089	0
P:/osteosa	1	0.978721	0.76349	0.38337	0.123801
P:/osteosa	1	0.970294	0.684323	0.080107	0.025526
P:/osteosa	1	0.938057	0.664735	0.280174	0
P:/osteosa	1	0.968552	0.768017	0.287143	0
